# Effects of an Unstructured Free Play and Mindfulness Intervention on Wellbeing in Kindergarten Students

**DOI:** 10.3390/ijerph17155382

**Published:** 2020-07-26

**Authors:** Regina Lai Tong Lee, Shelly Jerrine Lane, Anson Chiu Yan Tang, Cynthia Leung, Lobo Hung Tak Louie, Graeme Browne, Sally Wai Chi Chan

**Affiliations:** 1 School of Nursing and Midwifery, Faculty of Health and Medicine, University of Newcastle, Callaghan 2308, Australia; graeme.browne@newcastle.edu.au; 2 College of Health and Human Sciences, Colorado State University, Fort Collins, CO 80526, USA; Shelly.Lane@colostate.edu; 3 School of Nursing, Tung Wah College, Hong Kong, China; ansontang@twc.edu.hk; 4 Department of Social Sciences, The Hong Kong Polytechnic University, Hong Kong, China; ssleung@polyu.edu.hk; 5 Department of Physical Education, Hong Kong Baptist University, Hong Kong, China; s62591@hkbu.edu.hk; 6 University of Newcastle, Callaghan 2308, Australia; sally.chan@newcastle.edu.au

**Keywords:** preschool, kindergarten, unstructured play, loose parts play, mindfulness, physical, social and emotional wellbeing

## Abstract

Play is known as the core occupation of young children as it lays a foundation for their early development and physical, emotional and social wellbeing. Literature suggests that unstructured free play and mindfulness interventions may independently promote wellbeing among preschoolers. However, there is no clear evidence of their combination in supporting wellness in early learning environments. We conducted a quasi-experimental study with 42 children aged four to six years, attending two kindergartens in Hong Kong. The intervention included unstructured play with non-directional loose parts (play materials), conducted outdoors for one hour daily followed by a mindfulness intervention for 10 min per day indoors. The intervention lasted for five consecutive days. We examined happiness and aspects of playfulness before and after the intervention, finding a significant increase in all areas. Given greater freedom in play choice, children showed more disruptive behaviors during unstructured play than the control group engaging in recess as usual. We conclude that unstructured play in addition to mindfulness intervention is effective in promoting students’ happiness and playfulness, both of which may help maintain mental health and wellbeing amid stressors such as transition and separation. The increased disruptive behavior requires additional investigation.

## 1. Introduction

Play is known as the core occupation of young children, laying a foundation for their early development and physical, emotional and social wellbeing [1,2]. Play potentially reduces childhood obesity, peer interaction problems and mental health issues [3]. Children’s wellbeing has a high priority in healthcare and early childhood education, in the context of reducing the increasing number of children experiencing mental health problems as well as developmental, emotional and behavioral issues [4,5]. Through play, children develop multiple skills including those related to social interaction, communication, peer interaction, executive function, and problem-solving skills [1,2]. Children use play to work through experiences and events and renew their understanding of their changing world [6]. Child-directed, unstructured outdoor play offers an opportunity for young children to explore the natural environment and engage with their peers [3]. Outdoor free play allows the testing of physical limits, expressing oneself freely, building self-confidence and facilitating socialization, which may not be achieved through play with electronic games [3,7].

Spending time in preschool or school may be linked to low levels of physical activity [8]. As such, providing the opportunity to engage in physical activity and reducing sedentary time represents another potential benefit for play [4]. Investigations focusing on varying access to free play times in child care environments have shed light on the complexity of optimal free play time. Razak, et al. [9] worked with childcare facilities, serving children aged three to six years, to optimize unstructured play opportunities. Rather than the routine single 60-min block of free play, three shorter play periods (two morning periods of 15 min; one 30 min afternoon period) were offered to the children. Using a cluster randomized trial design (cluster RCT), the amount of moderate to vigorous physical activity (MVPA) in children during play was examined, looking at play as usual environments compared to MVPA in children in the intervention environment. Findings indicated that mean MVPA was greater in the intervention group, as was the overall percentage of time spent in MVPA. Somewhat in contrast, other investigators have introduced four 30-min free play sessions in preschool or child care environments, finding no positive impact on MVPA. Driediger, et al. [10] carried out a cluster RCT, providing the intervention group four 30-min periods of outdoor unstructured play daily for eight weeks. They failed to find a significant difference in physical activity between groups across time. Importantly, in this study compliance in wearing the accelerometer was poor and attrition high, resulting in the loss of nearly 30% of participant data. A similarly designed study, comparing four 30-min outdoor free play periods to a control of two periods per day, also failed to find between group differences [11]. However, this later study utilized a two-day baseline and two-day intervention period. In this very short duration intervention investigators found that there were no significant between-group differences in the changes in step counts per minute or time spent on MVPA during the school day. This study used a small sample size and the very short intervention period, and as such these findings should be viewed with caution.

Wolfenden, et al. [12] investigated the benefits of unrestricted access to outdoors for free play throughout the day, when structured activity was not taking place; this study also took place in childcare settings, using a cluster RCT design. Data was collected at baseline and at a three-month follow-up time point. However, in this study no significant between-group differences were found for minutes of MVPA) or total physical activity while in childcare. Investigators suggested that simply providing unrestricted access to outdoor free play was insufficient to encourage all children to engage in physical activity; some children preferred indoor activities. Furthermore, because all children were not engaged outside at the same time, investigators proposed that staff involvement during free play time involved fewer prompts and positive comments in the intervention group, something that was not seen in the comparison group. This might also have altered outcomes. Thus, for young children, scheduling several short opportunities for play may best support engagement in outdoor activities for the child, and optimize support from childcare staff.

Based on a pilot study using loose parts play materials, in which investigators found that children who had opportunities to engage with these materials during recess were perceived as more social, creative, and resilient [9], Bundy, et al. [1] developed a cluster RCT of unstructured play among children aged five to seven years in schools. They aimed to engage children’s natural playfulness instincts to promote physical activity and enhance the development of social skills. These investigators documented increased physical activity and argued that opportunities to experience the benefits of motor, social and emotional challenges could be offered to children through play activities on the school playground [5]. Expanding on this work, Engelen, et al. [13] carried out a cluster RCT, altering available play materials for the intervention classrooms and addressing adult concerns about risk-taking during playground play. Although all children had recess for one hour each school day, the intervention group was provided a variety of materials considered ‘loose parts’ play. Play materials included such things as swim noodles, old tires, stacking crates, large foam-filled pillows or mats, large pieces of foam and foam shapes, and large cardboard barrels. Paralleling the play intervention, investigators addressed teachers’ and parents’ concerns about play with loose parts materials in a joint workshop. The combination of play opportunity for the children and risk reframing for the adults involved was considered crucial because adult concerns about risk could circumvent child access to the play materials. Investigators found that the intervention group had significant increases in total step counts and duration of MVPA and a decrease in sedentary activity during break times when gender, school grade, body mass index, socioeconomic status and the size of play area were statistically controlled. It is notable that the frequency of intervention was higher (5 days/week) and the duration of intervention was longer (13 weeks) than other similar trials, which may have positively influenced findings.

In many of these studies missing data was a potential problem. Some investigators [8,10,13] opted not to include data suggesting inaccurate activity counts (e.g., a zero activity count for a period of ten minutes; records of less than 50 to 80% of the intended wear time per day; reduced wear time per week) The percentages of missing data were comparably high in both the shorter and the longer observation periods [10]. In two studies [9,12] multiple imputations to replace missing data was used, even when they had fewer missing data. Thus, missing data appears to be a factor that must be carefully considered.

Additional considerations in these studies included activity recording time. Some studies recorded children’s steps within the assigned intervention period and the setting of school or day care center [3,9,12,13], while others measured total steps during wake time across the entire day [8,11]. Studies also differed in sample size and age. Given the between studies differences it is difficult to draw any firm conclusions regarding whether providing opportunities for unstructured play intervention would improve the overall physical activity level in children’s daily life. Furthermore, it is not clear how best to structure access to such play materials, and whether this might differ depending on the age of the child. As such there is much yet to be done in this area.

While some evidence exists of the benefits of unstructured play, and perhaps even more relative to loose parts play, other interventions also show promise. In particular, mindfulness interventions may help young children control expression of emotions, as well as maintain social and emotional wellbeing. Mindfulness is described as the most basic forms of meditation [14]. It is process and quality of mind that guides us to be in the moment, to notice things as they take place and pass no judgement, involving noticing the mind-wandering and bringing attention back to an identified point of focus [15]. Mindfulness training may help children develop many skills, including self-regulation and attention [15,16] and is reported to support improvements in learning and health [15]. The study of mindfulness training for children has gained interest, and while more research is needed, outcomes are promising [15,16,17]. Mindfulness training, often coupled with other interventions, has been shown to positively impact learning, social competence, social-emotional development, and various executive functions [15,16,17]. For instance, Flook, et al. [15] carried out a cluster RCT on 68 elementary schoolchildren, mean age five years, nested within seven classrooms. A mindfulness-based kindness curriculum (20 to 30 min, ×2/week, for 12 weeks) was provided to the intervention group and outcomes compared to a wait-list control group. Children receiving the mindfulness curriculum had better marks at the end of the school year in the domains of learning, health, and social-emotional development. In addition, teacher-rated social competence, as well as the prosocial behavior and emotion regulation, were better for the intervention group. In spite of relatively small size of the clusters and non-blinded raters, this intervention has merits.

Poehlmann-Tynan, et al. [17] also used a cluster RCT design to investigate the feasibility of implementing a mindfulness intervention with economically disadvantaged preschool children. The intervention group received one-hour of a mindfulness intervention in addition to a three-hour dialogic reading program per week for 12 weeks. Compared with the control group, children in the intervention group significantly increased their attentional focus and self-regulation skills from baseline to post-test. Moreover, the improvement in self-regulation was maintained at follow-up. The data collectors were blinded to the group assignment, but the samples size of the clusters was small. Other literature also documents that even a guided mindfulness intervention as brief as three to four sessions of five to 10 min can buffer affective reactivity and reduce impulsive behaviors among young children [12,13,14,15,16,17,18,19,20,21].

It has been reported that periods of transition, for instance in transitioning to school from kindergarten, can be challenging for young children. For instance, parents report that children have difficulty with the different approaches to teaching and teachers report challenges with children following rules [22]. Furthermore, children report wishing to return to kindergarten, where they had experienced less pressure to perform [23]. Moreover, children are reported to experience an increase in stress that may last as long as six months into the school year [24] and limited their ability to successfully use coping strategies [25]. Stressful transitions have been noted to potentially impair the learning process and childhood development [25].

Thus, there are potential benefits of both unstructured/loose parts play and mindfulness interventions; no studies were identified that incorporated both. The aim of this study was to examine the effectiveness of unstructured loose parts play in addition to a mindfulness intervention in promoting physical activity level, emotional wellbeing, peer interaction and playfulness among preschoolers compared with those who underwent usual play at the kindergarten.

The hypothesis of the current study was that, compared to a control group engaging in recess as usual, physical activity level, emotional wellbeing, peer interaction and playfulness would be significantly better among preschoolers who played outside with unstructured loose parts materials during recess and engaged in mindfulness activities throughout the week-long intervention period.

## 2. Methods

### 2.1. Design

The current study used a parallel two-arm quasi-experimental design. Two kindergartens were recruited, one assigned as intervention and the other a comparison group. A pre-intervention assessment was conducted the week prior to implementation of the intervention. The intervention was conducted for five consecutive days in March 2019, and a post-intervention assessment was conducted the week following intervention. There was no change to methods after the commencement of the study.

### 2.2. Participants

Kindergartens and students were convenience sampled. The principal investigator (PI) met and provided project details to the two kindergartens’ principals and the schoolteachers with the information sheet and consent form for parents. After seeking approval from the management committee of two kindergartens, the two researchers delivered briefing sessions to the parents arranged by the kindergarten principals in one of the classrooms. The parents returned the consent forms through the kindergartens’ communication system. We only recruited those children who met the inclusion criteria with the returned parental consent forms. The two kindergartens included were those (1) registered under the Hong Kong Education Bureau, (2) having a play area for intervention and observation, and (3) having at least one hour for lunch time and recess for students each school day. The inclusion criteria of students were those who were (1) aged between three and seven years, and (2) typically developing with no physical or developmental disabilities. Data were collected for control and intervention groups within the environment in which recess took place. For the control children data were collected in an indoor assembly hall. For the intervention group data were collected in the indoor activity area at baseline, and on the outdoor playground outside at post-test.

### 2.3. Intervention

We used a multidisciplinary approach to develop our intervention program, capitalizing on skills and knowledge from community health nursing, occupational therapy and early childhood education. The program, Supporting Wellness in Early Learning Environments (SWELE) combined loose parts free play and mindfulness sessions. SWELE offered children the opportunity to explore and strategize, develop interaction and cooperation skills, build peer relationships during unstructured free play, and engage in activities designed to reduce anxiety and increase focus and attention during mindfulness sessions.

In this study unstructured free play involved providing children access to the loose parts play materials that have no specific purpose. We included a myriad of play materials, including such items as paper boxes, hula hoops, cones, bean bags, car tires, tree sticks, tree leaves, strawman, paper sticks, blank paper, paint, and paint brushes. The materials used were the same on each intervention day and were made available in the outdoor playground. Play lasted 45 min each day.

In this study, the mindfulness intervention involved having children sit quietly inside a room. The PI then led the children through storytelling, deep-breathing exercises, and body scanning. The PI asked the children to imagine the air going to a different part of the body, to notice the sensations such as tingling, warmth, coolness or pressure in that part of body, and then to exhale any those of uncomfortable sensations they may be feeling in that part of body. The activities followed a reproducible sequence each day. Mindfulness activities were undertaken for 15 min daily, indoors, and immediately following play. The control group engaged in recess as usual, following their established routine. These children played with toys and fixed equipment indoors, in the assembly hall of the school. The total play time for the control group was approximately 60 min as there were no mindfulness activities.

The PI led the children through storytelling for deep-breathing exercises, body scanning and being aware of their thoughts and emotions. Each session was divided into a series of three mindfulness exercises, which focused on three types of activities: (i) mindfulness of breathing, (ii) mindfulness of body, (iii) and mindfulness of thoughts and emotions. All mindfulness activities were offered to children as “story-telling” to create mental images, meant to promote awareness of the three aspects of the self, related to breath, body, and thoughts/emotions. In each of the sessions, children were first required to concentrate on breath whilst refraining from actively controlling it. In the second activity, they had to concentrate their attention on different body parts. In the last activity, children were encouraged to observe the stream of their thoughts and emotions through storytelling. For example, an apple on the belly was used to better focus and observe the breath, while imaging thoughts such as soap bubbles, sea waves or clouds was believed to help children experiencing and understanding the transitory process of thoughts. During the body contemplation exercises, mindful movement was used such that children were asked to mindfully explore their body while walking and imagining that the floor was made of sand or grass.

The PI explained to the children on the three mindfulness activities in the language that they understood through storytelling. The children were told that the purpose of mindfulness activities was to make the body relax and they feel better. The PI had also checked the children’s understanding by asking “Could you tell me why we are now doing the mindfulness activities?” The children were able to state the reasons for doing the mindfulness activities.

### 2.4. Outcomes

The instruments for data collection included questionnaires for parents, pedometers, stadiometers, weight scales, and psychometric scales for children. Prior to beginning child-based data collection, parents completed the parent questionnaires at the kindergartens. The questionnaire for parents included items of date, child’s name, and parents’ education level. The assessors also asked the children about their demographic information and recorded the responses. Children were assigned a research code constructed from an abbreviated name and the child’s initials. Demographic information relative to child name, age, gender and grade were recorded.

#### 2.4.1. Measures of Physical Wellbeing

An electric pedometer, Yamax Digi-Walker CW-701, was used to count the children’s steps during play. The pedometer was attached onto each child’s trousers at the right iliac crest during play at pre-test and post-test. This device collects data relative to step counts, distance and calories. The concurrent validity of CW-701 was satisfactory [26]. No significant differences were identified in the step count for both groups in this study.

Additional measures of physical wellbeing included weight and calculation of body mass index (kg/m^2^). At pre-test and post-test, children’s height (cm) and weight (kg) were measured by the assessors using stadiometers and recorded before and after the intervention.

#### 2.4.2. Emotional Wellbeing

The Smiley Face Likert Scale was used to assess children’s level of happiness after play [27]. Each child was asked to choose the face on the Smiley Face Likert Scale which reflected his or her level of happiness after play. There were eight items which evaluated the easiness, funniness, excitement and method of play; children’s desire to play again and for longer; and the appearance and accessibility of the play environment. The smileyometer was a five-point Likert scale ranging from “1 = wow (greatest smile)” to “5 = oh dear (no smile)”. The scores were reversed, therefore higher score indicates higher level of happiness. Although children completed the Smiley Face scale daily following play, in this study we used only pre-test and post-test ratings.

The Children’s Emotional Manifestation Scale [28] classifies observable emotional behaviors of children during play. This scale was completed at pre- and post-test time points by the trained raters and included interviews with each child. The scale contains five items which are facial expression, vocalization, activity, interaction, and level of cooperation. Each item is measured on a five-point scale from “1 = positive emotion” to “5 = negative emotion”. The content validity (CVI = 0.96) and convergent validity with the State Anxiety Scale (*r* = 0.76, *p* < 0.01) are satisfactory. Moreover, the internal consistency (Cronbach’s alpha = 0.92) and inter-rater reliability (ICC = 0.96) are excellent. The scores are reversed, therefore higher scores indicate more positive emotions.

#### 2.4.3. Social Wellbeing

The Penn Interactive Peer Play Scale was used to assess the child’s behaviors, competencies, needs, success and difficulties with peers during play [29]. This tool was designed for use with children in preschool and kindergarten and includes parallel parent and teacher versions. Each of the 36 items are measured on a four-point scale from “1 = never” to “4 = always”. Factor analysis and canonical analysis has supported a three-factor structure, termed dimensions [30]. This tool has acceptable internal consistency for each dimension (Cronbach’s alpha = 0.74–0.84) and interrater reliability assessment revealed a significantly high correlation of 0.88 (*p* < 0.001) [30]. The three dimensions defined by this tool include Disruption (10 items), Disconnection (nine items) and Interaction (10 items). Disruption reflects behaviors such as aggression and antisocial play behaviors. Disconnection reflects not participating in play, evidenced by withdrawing, wandering, hovering, and being ignored by playmates. The third dimension, Interaction, is characterized by sharing ideas, helping and encouraging other children to engage in play, and leading [30]. Scoring for items belonging to Disruption and Disconnection are reversed, therefore higher scores indicate less behavior problems.

The Test of Playfulness Scale was used to assess the child’s disposition to engage in play [31]. This scale consists of three domains of playfulness: the extent of time the behavior was observed (eight items), the intensity of the behavior (five items), and the child’s skillfulness in demonstrating the behavior (16 items). Scores for each scale are summed for a total Playfulness score. Each item is measured on a four-point scale from “0 = lowest level” to “3 = highest level”. Using Rasch analysis, the scale has been shown to measure the overall construct of playfulness. In addition, 98% of the children showed valid person-response patterns and all raters fit the measurement model [32]. Higher scores reflect higher level of playfulness. For this investigation raw scores for each domain of playfulness were used. In this study children’s play activities were recorded for 20 min each day, and playfulness was scored from the videotapes, as is recommended for the Test of Playfulness. The videos from the pre-test and post-test were rated by trained raters at the university and used in this study.

### 2.5. Sample Size

A targeted sample size was calculated using G power 3.1.9.2 2 (Heinrich-Heine-Universität, Düsseldorf, Germany). The significance level was set at 0.05 and the power was set at 80 per cent. Assuming a small to moderate effect size of 0.25 and a correlation coefficient of 0.5 between repeated measures, the total sample size required was 34 for an F test of group by time interaction. Assuming a consent rate of 90% and an attrition rate of 10%, the required sample size was 42. Therefore, a minimum of 21 students from each kindergarten was required.

Each kindergarten in this study had 30–35 students. Because we required individual videotapes of children playing and this mandated that a research assistant tracked one child throughout the 45–50 min play time, we restricted our enrollment to the required sample size of 42, including the first 20–22 children in each kindergarten from whom we received parent questionnaires and consent forms.

### 2.6. Group Allocation

Two kindergartens were randomized into an intervention and control group with the use of randomizing software. The allocation ratio was 1:1. Opaque envelopes contained the results of group allocation and were delivered to each kindergarten by a research assistant. The interventionist (PI), assessors and the kindergarten teacher were concealed from the allocation results but opened the envelopes on the first day of the intervention period. The kindergarten teacher enrolled children and parents based on convenience sampling.

### 2.7. Blinding

The research assistant, kindergarten teacher, assessors and raters of videos were blinded to the study hypothesis. In addition, the two raters of videos were blinded to the time points at which the videos were taken. The interventionist (PI) could not realistically be blinded to the study hypothesis or group assignment. However, the interventionist was not involved in assessments. Parents were informed about the group allocation by the end of the intervention period.

### 2.8. Statistical Methods

IBM SPSS Statistics for Windows, Version 21.0. (IBM Corp., Armonk, NY, USA) was used to analyze demographic data with chi-squared tests. The SAS University Edition (Institute Inc., Cary, NC, USA) was used to fit data of physical outcomes and psychometric scales in regression models. The probability distribution of the response was assumed as normal, thus identity link function was specified. The fixed effects were age, gender, group, time and group by time interaction. The parameters were estimated by using the method of residual marginal pseudo-likelihood. The estimation of the standard errors was adjusted with a bias-corrected covariance estimator developed by Mancl and DeRouen [33]. The covariance structure between repeated measures was specified as first-order autoregressive. The p values in multiple comparisons of least squares means were adjusted with Dunnett’s [34] method. The significance level was set at 0.05. The analysis followed the intention-to-treat principle.

### 2.9. Ethical Considerations

Ethical approval was obtained from the University of Newcastle Human Research Ethics Committee, 26 March 2019. Research proposal approval number is H-2018-0335. Project flyers, information statements and consent forms were distributed to the principals, teachers, and parents via the school communication system in each kindergarten. The information sheet and consent form explained the study purpose, brief procedure, potential risks, confidentiality and the voluntariness of participation with no penalty for withdrawal. Informed consents were obtained from all parents prior to the commencement of the study. Data were only stored, accessed and analyzed by the researchers.

## 3. Results

Twenty children were allocated to the intervention group and 22 children were in the control group (see Table 1). Age and gender distribution did not differ between groups. At pre-test there were two children absent in each group. There were between group significant differences in parents’ education level. Approximately 60% of the parents completed secondary school, while nearly 30% of the parents completed tertiary education. There was <5% missing data overall, although missing data for pedometer readings was slightly higher, at 7%.

Table 2 shows the regression coefficients and the 95% confidence intervals of body mass index, step count, happiness score and emotion score. Only the happiness score shows significant group by time interaction effect. However, the group effect was not significant. The happiness score significantly increased within the intervention group (mean difference = 0.67, 95% confidence interval (CI) = 0.3 to 1), and the score within control group did not change (see Table 3). Notwithstanding that, the score in the control group was higher than the intervention group at baseline.

Table 4 shows the results of the domain scores of interactive peer play and playfulness assessments. There were significant group, time and group by time interaction effects on the Disruption domain. The disruptive behaviors became significantly more severe within the intervention group (mean difference = −0.26, 95% CI = −0.4 to −0.12) but not in the control group (see Table 3). Furthermore, the disruption in intervention group was significantly more severe than control group at post-test (mean difference = −0.21, 95% CI = −0.4 to −0.023), given that there was no baseline difference. We had hypothesized that the physical activity level, emotional wellbeing, peer interaction and playfulness would be significantly better among preschoolers in the intervention group. Findings supported increased playfulness and improved emotional wellbeing in the group of children engaging in outdoor, loose-parts play and short mindfulness sessions.

On the other hand, the group, time and group by time interaction effects in all domains of playfulness were significant (see Table 4). The playfulness of the intervention group was significantly better than the control group at post-test even though there were significant baseline differences (see Table 3). Nevertheless, the improvements in the extent, intensity and skill of play within the intervention group were larger than the control group, provided that the changes in both groups were significant except for the skills in the control group.

## 4. Discussion

### 4.1. Interpretation

We had hypothesized that the physical activity level, emotional wellbeing, peer interaction and playfulness would be significantly better among preschoolers in the intervention group. Findings supported increased playfulness and improved emotional wellbeing in the group of children engaging in outdoor, loose parts play and short mindfulness sessions. However, we did not find group differences for physical activity level or peer interaction.

Although both groups showed changes in playfulness between pre- and post-test, the intervention group was significantly more playful than the control group at post-test, and the improvement in playfulness within intervention group was larger than the control group. The findings are similar to other studies on evaluating the changes in playfulness [35,36]. Brentnall et al. found that there were significant differences in each observational rating score in the playfulness scale on pre-school age children’s play activities [35]. Bronson and Bundy investigated the correlation between playfulness and environmental supportiveness between children with disability and typically developing children [36]. They reported that there was a positive correlation between playfulness and environmental supportiveness especially among the typically developing children.

A finding unique to this study was that the happiness score significantly increased within the intervention group, while the score within the control group did not show any change. It is difficult to tease out the driver for this change as it could be linked to both playfulness and mindfulness. Badri et al. [37] conducted a study to investigate the structural relationship such as school and home environments on school children’s happiness. The findings showed that both school and home variables directly influence the school children’s happiness scores [37].

We had hypothesized that the emotional wellbeing would be significantly better among preschoolers in the intervention group. There were no significant differences in emotion scores as the scores in both groups were high at all time points. It may perhaps be because of insufficiently long sessions or insufficient overall intervention. There was change in happiness scores, which is likely to reflect the impact of mindfulness activities and play opportunity in the intervention group. Interestingly, disruptive behaviors increased within the intervention group, and disruption in the intervention group was more severe than the control group at post-test. This is perhaps not surprising given that typical play during recess for children in this study was highly controlled and structured. The more gregarious and free play notable for children given the freedom to engage in play without structure might have been interpreted by the trained raters as more disruptive. Our findings are consistent with the findings of Trostle [38]. Children in this study participated in either behavioral intervention or unstructured play across a 10-week intervention. The behavioral group showed a greater improvement in self-control than the unstructured play group. These findings suggest that there may be lower self-control and behavior may be viewed as more disruptive when children engage in unstructured play. In fact, the correlation between self-control and disruptive behavior among children has been identified in other investigations [39,40,41].

Our findings support, to some extent, what has been identified in previous studies [3,30,35,36,37]; engaging in loose parts play and mindfulness can have a positive impact on playfulness and children’s happiness. The absence of significant change on the physical variables such as step count and body mass index in the current study might be attributable to the short intervention period. Although the sample size was larger and the intervention period was longer than the study conducted by Alhassan, Sirard and Robinson [11], it was nonetheless relatively short and considerably shorter than that shown by other investigators [13].

The significant baseline difference in the step count is likely related to the differences in the shape and size of the play area between kindergartens. Other investigations have suggested that available space may influence the nature of the play activities [11,13]. In the current study the intervention group pretesting was conducted outdoors in a relatively large oval area. Children had space to move and play and appeared to take advantage of this. In contrast, the control group played with usual toys and fixed equipment indoors, in a more confined rectangular area, throughout the study. Interestingly the step count for intervention children increased from pre- to post-testing, while that for the control group decreased. This suggests that recess as usual was not supportive of child physical activity, something known to be quite important for learning [42,43]. Hence, the physical activity level of the control group children might be lower than the intervention group children who ran around in the oval area.

Previous studies of mindfulness intervention which significantly improved social and emotional development, social competence, prosocial behavior and emotion regulation [15] and self-regulation [17] of young children implemented the intervention for around 20 to 30 min per session, one hour per week for 12 weeks. In the current study, the session lasted only for 15 min per day for five days, and the intervention group showed significantly more disruptive behaviors during unstructured play than the control group. In the future study, the session shall be as long as the one in the previous studies so as to draw a conclusion on whether the mindfulness intervention could promote behavioral control during unstructured play or not. Based on the results in this study, 15 min per day for five days might fall short of promoting behavioral control during unstructured play among young children.

### 4.2. Limitations

Limitations inherent in these findings include the fact that time and budget limited the sample size and study period. While there is value in short intense interventions, other studies have shown additional gains with longer interventions. The sample size of the clusters was small for randomization, and while this may be associated with the baseline differences, change scores support an intervention effect. Furthermore, the test-retest reliability of assessment may change over time. Test-retest reliability of playfulness could become moderate to poor when the duration of rating increases from 15 to 30 min [35]. Still, 15–20 min is the recommended amount of time for accurately rating playfulness. On the other hand, the insignificant change in physical activity level may be related to the short intervention and observation period as well as insufficient statistical data on unstructured play and mindfulness sessions, and we are not able to distinguish the benefits of each component separately. Despite the limitations, this study provided pilot data and directions for future bigger scale studies.

### 4.3. Generalizability

The convenience sampling, small sample size and risk of bias in the current study limit the generalizability of the results. However, this work indicates that, at least in some preschool environments, has the potential to implement an outdoor, unstructured play activities in the school setting. This work, combined with that of Bundy and colleagues, suggests the potential to further develop the intervention as a community health intervention. The unstructured play activities and mindfulness activities for promoting children’s wellbeing may be adopted to change health policy and practice targeting early childhood to promote ‘Healthy Children, Happy Learning’. Preschools and primary schools may translate the evidence into school health practice leading to changes in physical health and social wellbeing of preschoolers.

### 4.4. Future Researce

In future studies, a longer intervention period should be adopted, and the sample size of the clusters should be larger for a cluster RCT. A waitlist control group should also be considered so that all the participants will receive and benefit from the intervention. Besides comparing loose part and fixed equipment for play, future studies may compare play using different play materials to support unstructured play; choice of materials should be linked to that which is most feasible given different environments [44]. Furthermore, variables such as the time periods of play and observation, as well as the shape and size of the play area per child should be assessed and analyzed. In addition, a focus on mindfulness, defining optimal content, duration, and timing of sessions during the day would clarify the contribution of this aspect of our study. Finally, factorial design including unstructured play, mindfulness intervention and their combination may be adopted to differentiate the effect of each arm statistically.

### 4.5. Implications

Children often find the transitions from home, to preschool and then to schooling distressing [22] despite the fact that some may look forward to a shift in emphasis from play activity to structured learning [45]. Stressful transitions can interfere with the learning process and even childhood development [25]. It has been reported that 22% of Australian children experience difficulty during their transition to school in at least one of the following domains: physical health and wellbeing, social competence, and emotional maturity [46].

Incorporating opportunities for unstructured outdoor play and providing mindfulness activities could facilitate a smooth transition process. Through play and self-reflection, children strategize about change and transition which characterize early childhood. Opportunities to develop skills and problem solving through unstructured play are increasingly limited as children prepare to enter school and the emphasis turns to structured learning in classroom setting. The limitations to children’s free play deserve more research and public attention because it may have negative impacts on children’s physical and psychosocial wellbeing. Mindfulness, while promising, is not well integrated into the school day for most children.

## 5. Conclusions

Affording children, the opportunity for playful recess has been viewed as important for many years [47,48]. Here we expand on traditional school recess, to include interaction with loose parts play and engagement in mindfulness activities. The combination of these interventions offered several potential benefits to children in this study. In examining physical, emotional, and social wellbeing we identified increased physical activity (contributing to physical wellbeing), happiness (contributing to emotional wellbeing), and playfulness (contributing to social wellbeing) following this combined intervention. In this study also reported increased disruptive behavior during play. While this later finding needs further investigation, it is likely an outcome of greater physical freedom in a larger space during recess. Other findings support the conclusion that unstructured play plus mindfulness interventions benefit preschoolers’ overall wellbeing by providing them with materials, time, space and opportunities.

## Figures and Tables

**Table 1:** Socio-demographic characteristics of the young children and their parents (*n* = 42).

Characteristics	Categories	Intervention (*n* = 20)	Control (*n* = 22)	χ^2^	*p*
Gender	Male	9	12	0.38	0.56
	Female	9	8		
Age	4	12	10	1.68	0.43
	5	6	9		
	6	0	1		
Father’s education level	Primary	0	1	1.59	0.45
	Secondary	11	14		
	University	7	5		
Mother’s education level	Primary	0	1	3.5	0.17
	Secondary	10	16		
	University	8	4		

**Table 2:** Regression coefficients of body mass index, steps count, happiness and emotion.

Term	Body Mass Index	Steps Count	Happiness	Emotion
	B [95% CI]	B [95% CI]	B [95% CI]	B [95% CI]
Intercept	15.7 [15.4, 16.1] *	2384.1 [2138.4, 2629.8] *	4.4 [4.3, 4.6] *	4.8 [4.7, 4.9] *
Age	0.55 [−2, 3.1]	−1897.5 [−4221, 426]	−0.7 [−1.9, 0.5]	0.3 [−0.26, 0.85]
Male	−0.3 [−17.6, 17]	2381 [−12478, 17240]	−0.22 [−7.3, 6.8]	0.31 [−3.4, 4]
Female	0	0	0	0
Control	3 [−0.39, 6.3]	−7582.4 [−10828, −4336.9] *	−0.55 [−1.9, 0.84]	0.18 [−0.74, 1.1]
Intervention	0	0	0	0
Pretest	−0.39 [−2.5, 1.7]	−180.4 [−2858.7, 2498]	−3 [−4.6, −1.3] *	0.42 [−0.31, 1.2]
Posttest	0	0	0	0
Control by pretest	0.084 [−2.4, 2.5]	968.1 [−1647.8, 3584]	2.5 [0.64, 4.3] *	−0.16 [−1.1, 0.78]
Control by posttest	0	0	0	0
Intervention by pretest	0	0	0	0
Intervention by posttest	0	0	0	0

* *p* < 0.05.

**Table 3:** Least squares means of physical and psychosocial variables between groups across time.

Outcome	Time point	Intervention	Control	Difference, between
		Mean [95% CI]	Mean [95% CI]	Mean [95% CI]
Body mass index	Pretest	15.3 [14.9, 15.8]	16 [15.4, 16.7]	−0.69 [−1.4, 0.07]
	Posttest	15.4 [14.9, 16]	16.1 [15.5, 16.7]	−0.66 [−1.4, 0.092]
	Difference, within	0.087 [−0.38, 0.55]	0.065 [−0.38, 0.51]	
Steps count	Pretest	3139.3 [2809.8, 3468.7]	1677 [1253.4, 2100.6]	1462.3 [911.3, 2013.2] *
	Posttest	3180.2 [2530.5, 3829.8]	1462.6 [1155, 1770.1]	1717.6 [976.7, 2458.6] *
	Difference, within	40.9 [−566.4, 648.2]	−214.5 [−559.5, 130.5]	
Happiness	Pretest	4 [3.7, 4.3]	4.5 [4.2, 4.8]	−0.52 [−0.93, −0.099] *
	Posttest	4.7 [4.5, 4.8]	4.5 [4.3, 4.8]	0.12 [−0.19, 0.44]
	Difference, within	0.67 [0.3, 1] *	0.03 [−0.25, 0.31]	
Emotion	Pretest	4.8 [4.7, 4.9]	4.8 [4.7, 4.9]	0.0019 [−0.16, 0.16]
	Posttest	4.7 [4.6, 4.9]	4.8 [4.6, 4.9]	−0.04 [−0.25, 0.17]
	Difference, within	−0.095 [−0.26, 0.07]	−0.053 [−0.23, 0.12]	
Disruption	Pretest	4 [3.9, 4]	4 [3.9, 4]	−0.0045 [−0.053, 0.044]
	Posttest	3.7 [3.5, 3.9]	3.9 [3.8, 4]	−0.21 [−0.4, −0.023] *
	Difference, within	−0.26 [−0.4, −0.12] *	−0.049 [−0.15, 0.05]	
Disconnection	Pretest	3.8 [3.6, 3.9]	3.9 [3.8, 4]	−0.091 [−0.26, 0.082]
	Posttest	3.6 [3.4, 3.8]	3.8 [3.6, 3.9]	−0.15 [−0.42, 0.11]
	Difference, within	−0.17 [−0.31, −0.023] *	−0.1 [−0.28, 0.078]	
Interaction	Pretest	1.1 [0.77, 1.4]	0.96 [0.69, 1.2]	0.11 [−0.3, 0.53]
	Posttest	1.3 [0.99, 1.6]	0.76 [0.41, 1.1]	0.56 [0.071, 1] *
	Difference, within	0.24 [−0.1, 0.59]	−0.21 [−0.67, 0.26]	
Play extent	Pretest	1.5 [1.4, 1.6]	1.3 [1.2, 1.4]	0.2 [0.058, 0.34] *
	Posttest	2.2 [2.1, 2.4]	1.5 [1.4, 1.6]	0.74 [0.51, 0.96] *
	Difference, within	0.78 [0.55, 1] *	0.24 [0.13, 0.35] *	
Play intensity	Pretest	1.3 [1.1, 1.5]	1.6 [1.5, 1.8]	−0.35 [−0.6, −0.1] *
	Posttest	2.3 [2, 2.5]	1.9 [1.8, 2]	0.36 [0.13, 0.59] *
	Difference, within	0.98 [0.76, 1.2] *	0.27 [0.084, 0.45] *	
Play skill	Pretest	0.43 [0.35, 0.51]	0.77 [0.71, 0.84]	−0.35 [−0.45, −0.24] *
	Posttest	1.6 [1.3, 1.9]	0.78 [0.71, 0.85]	0.83 [0.53, 1.1] *
	Difference, within	1.2 [0.9, 1.5] *	0.0065 [−0.074, 0.087]	

* *p* < 0.05. Score ranges: Happiness (1–5), Emotion (1–5), Interactive peer play (disruption, disconnection, interaction) (1–4), Playfulness (extent, intensity, skill) (0–3).

**Table 4:** Regression coefficients of peer interaction during play and playfulness.

Term	Disruption	Disconnection	Interaction	Play Extent	Play Intensity	Play Skill
	B [95% CI]	B [95% CI]	B [95% CI]	B [95% CI]	B [95% CI]	B [95% CI]
Intercept	3.9 [3.8, 4] *	3.8 [3.7, 3.9] *	1 [0.87, 1.2] *	1.6 [1.6, 1.7] *	1.8 [1.7, 1.9] *	0.9 [0.82, 0.97] *
Age	−0.072 [−0.55, 0.4]	−0.35 [−1.2, 0.52]	0.87 [−0.93, 2.7]	−0.036 [−0.65, 0.58]	−0.02 [−0.82, 0.78]	0.22 [−0.51, 0.95]
Male	0.23 [−3, 3.4]	0.41 [−5, 5.8]	0.56 [−9.1, 10.2]	−0.22 [−3.8, 3.4]	−0.4 [−6.3, 5.5]	−0.47 [−5.3, 4.4]
Female	0	0	0	0	0	0
Control	0.96 [0.11, 1.8] *	0.69 [−0.49, 1.9]	−2.5 [−4.7, −0.33] *	−3.3 [−4.3, −2.3] *	−1.6 [−2.7, −0.58] *	−3.7 [−5.1, −2.4] *
Intervention	0	0	0	0	0	0
Pretest	1.2 [0.51, 1.8] *	0.74 [0.1, 1.4] *	−1.1 [−2.6, 0.47]	−3.5 [−4.6, −2.5] *	−4.4 [−5.4, −3.5] *	−5.4 [−6.6, −4.1] *
Posttest	0	0	0	0	0	0
Control by pretest	−0.81 [−1.5, −0.14] *	−0.25 [−1.2, 0.67]	1.7 [−0.52, 4]	2.2 [1.1, 3.2] *	2.9 [1.7, 4] *	4.7 [3.5, 5.9] *
Control by posttest	0	0	0	0	0	0
Intervention by pretest	0	0	0	0	0	0
Intervention by posttest	0	0	0	0	0	0

* *p* < 0.05.
