# Erratum: Lee, R.L.T., et al. Effects of an Unstructured Free Play and Mindfulness Intervention on Wellbeing in Kindergarten Students. *Int. J. Environ. Res. Public Health* 2020, *17*, 5382

**DOI:** 10.3390/ijerph18063163

**Published:** 2021-03-19

**Authors:** Regina Lai Tong Lee, Shelly Jerrine Lane, Anson Chui Yan Tang, Cynthia Leung, Stephen Wai Hang Kwok, Lobo Hung Tak Louie, Graeme Browne, Sally Wai Chi Chan

**Affiliations:** 1 School of Nursing and Midwifery, Faculty of Health and Medicine, University of Newcastle, Callaghan 2308, Australia; graeme.browne@newcastle.edu.au; 2 College of Health and Human Sciences, Colorado State University, Fort Collins, CO 80526, USA; Shelly.Lane@colostate.edu; 3 School of Nursing, Tung Wah College, Hong Kong, China; ansontang@twc.edu.hk; 4 Department of Applied Social Sciences, The Hong Kong Polytechnic University, Hong Kong, China; ssleung@polyu.edu.hk; 5 School of Nursing, The Hong Kong Polytechnic University, Hong Kong, China; waihang-stephen.kwok@polyu.edu.hk; 6 Department of Physical Education, Hong Kong Baptist University, Hong Kong, China; s62591@hkbu.edu.hk; 7 University of Newcastle, Callaghan 2308, Australia; sally.chan@newcastle.edu.au

The authors wish to make the following corrections to their paper [1].

1. The authors wish to replace: 

Regina Lai Tong Lee ^1,^*, **Shelly Jerrine Lane ^2^, Anson Chiu Yan Tang ^3^, Cynthia Leung ^4^, Lobo Hung Tak Louie ^5^, Graeme Browne ^1^ and Sally Wai Chi Chan ^6^**

^1^  School of Nursing and Midwifery, Faculty of Health and Medicine, University of Newcastle, Callaghan 2308, Australia; graeme.browne@newcastle.edu.au
^2^  College of Health and Human Sciences, Colorado State University, Fort Collins, CO 80526, USA; Shelly.Lane@colostate.edu
^3^  School of Nursing, Tung Wah College, Hong Kong, China; ansontang@twc.edu.hk
^4^  Department of Social Sciences, The Hong Kong Polytechnic University, Hong Kong, China; ssleung@polyu.edu.hk
^5^  Department of Physical Education, Hong Kong Baptist University, Hong Kong, China; s62591@hkbu.edu.hk
^6^  University of Newcastle, Callaghan 2308, Australia; sally.chan@newcastle.edu.au

with

Regina Lai Tong Lee ^1,^*, **Shelly Jerrine Lane ^2^, Anson Chui Yan Tang ^3^, Cynthia Leung ^4^, Stephen Wai Hang Kwok ^5^, Lobo Hung Tak Louie ^6^, Graeme Browne ^1^ and Sally Wai Chi Chan ^7^**

^1^  School of Nursing and Midwifery, Faculty of Health and Medicine, University of Newcastle, Callaghan 2308, Australia; graeme.browne@newcastle.edu.au
^2^  College of Health and Human Sciences, Colorado State University, Fort Collins, CO 80526, USA; Shelly.Lane@colostate.edu
^3^  School of Nursing, Tung Wah College, Hong Kong, China; ansontang@twc.edu.hk
^4^  Department of Applied Social Sciences, The Hong Kong Polytechnic University, Hong Kong, China; ssleung@polyu.edu.hk
^5^  School of Nursing, The Hong Kong Polytechnic University, Hong Kong, China; waihang-stephen.kwok@polyu.edu.hk
^6^  Department of Physical Education, Hong Kong Baptist University, Hong Kong, China; s62591@hkbu.edu.hk
^7^  University of Newcastle, Callaghan 2308, Australia; sally.chan@newcastle.edu.au

2. The authors also wish to replace:

**Author Contributions:** Literature review, R.L.T.L., S.J.L., S.W.C.C., A.C.Y.T., C.L., L.H.T.L., G.B.; sampling, R.L.T.L., S.J.L., S.W.C.C.; intervention administration, R.L.T.L., A.C.Y.T.; data collection, R.L.T.L., A.C.Y.T.; rater training, R.L.T.L., A.C.Y.T.; data cleaning and analysis, R.L.T.L., S.J.L.; manuscript writing, R.L.T.L., S.J.L., S.W.C.C., A.C.Y.T., C.L., L.H.T.L., G.B. All authors have read and agreed to the published version of the manuscript.

with

**Author Contributions:** Literature review, R.L.T.L., S.J.L., S.W.C.C., A.C.Y.T., S.W.H.K., C.L., L.H.T.L., G.B.; sampling, R.L.T.L., S.J.L., S.W.C.C.; intervention administration, R.L.T.L., A.C.Y.T., S.W.H.K.; data collection, R.L.T.L., A.C.Y.T., S.W.H.K.; rater training, R.L.T.L., A.C.Y.T., S.W.H.K.; data cleaning and analysis, R.L.T.L., S.J.L., S.W.H.K.; manuscript writing, R.L.T.L., S.J.L., S.W.C.C., A.C.Y.T., S.W.H.K., C.L., L.H.T.L., G.B. All authors have read and agreed to the published version of the manuscript.

The authors would like to apologize for any inconvenience to the readers caused by this error. The article will be updated and the original will remain on the webpage.
